# Improving GNSS Ambiguity Acceptance Test Performance with the Generalized Difference Test Approach

**DOI:** 10.3390/s18093018

**Published:** 2018-09-09

**Authors:** Lei Wang, Ruizhi Chen, Lili Shen, Yanming Feng, Yuanjin Pan, Ming Li, Peng Zhang

**Affiliations:** 1 State Key Laboratory of Information Engineering in Surveying, Mapping and Remote Sensing, Wuhan University, Wuhan 430079, China; Lei.wang@whu.edu.cn (L.W.); yjpan@whu.edu.cn (Y.P.); lisouming@whu.edu.cn (M.L.); fenix@whu.edu.cn (P.Z.); 2 Collaborative Innovation Center for Geospatial Technology, Wuhan 430079, China; 3 School of Geodesy and Geomatics, Wuhan University, Wuhan 430079, China; 4 Science and Engineer Faculty, Queensland University of Technology, Brisbane QLD 4000, Australia; y.feng@qut.edu.au

**Keywords:** GNSS, Ambiguity Resolution, Quality Control

## Abstract

In Global navigation satellite system (GNSS) data processing, integer ambiguity acceptance test is considered as a challenging problem. A number of ambiguity acceptance tests have been proposed from different perspective and then unified into the integer aperture estimation (IA) framework. Among all the IA estimators, the optimal integer aperture (OIA) achieves the highest success rate with the fixed failure rate tolerance. However, the OIA is of less practical appealing due to its high computation complexity. On the other hand, the popular discrimination tests employ only two integer candidates, which are the essential reason for their sub-optimality. In this study, a generalized difference test (GDT) is proposed to exploit the benefit of including three or more integer candidates to improve their performance from theoretical perspective. The simulation results indicate that the third best integer candidates contribute to more than 70% success rate improvement for integer bootstrapping success rate higher than 0.8 case. Therefore, the GDT with three integer candidates (GDT3) achieves a good trade-off between the performance and computation burden. The threshold function is also applied for rapid determination of the fixed failure rate (FF)-threshold for GDT3. The performance improvement of GDT3 is validated with real GNSS data set. The numerical results indicate that GDT3 achieves higher empirical success rate while the empirical failure rate remains comparable. In a 20 km baseline test, the success rate GDT3 increase 7% with almost the same empirical failure rate.

## 1. Introduction

In the global navigation satellite system (GNSS) data processing and GNSS based remote sensing applications, integer ambiguity resolution is an important and challenging research problem. The mathematical model for carrier phase based GNSS positioning model can be expressed as [1]:

(1) $$E(y)=Aa+Bb,D(y)=Q_{yy},a\in {\mathbb{Z}}^{n},b\in {\mathbb{R}}^{p}\;$$

where a and b are the integer and real-valued parameters, respectively. A and B are corresponding design matrices. The observation vector y follows m dimensional multivariate normal distribution with its variance-covariance (vc) matrix $Q_{yy}$. 

The mixed integer model can be solved by a four-step procedure [2]:

1. Estimating a and b using a least-squares estimator or Kalman filter. The integer nature of a is not considered in this step and corresponding estimated parameters are regarded as the ’float solution’. The float solution and its variance-covariance matrix are denoted as:
  (2) $$\left(\begin{array}{c} \hat{a}\\ \hat{b} \end{array}\right),\left(\begin{array}{cc} Q_{\hat{a}\hat{a}} & Q_{\hat{a}\hat{b}}\\ Q_{\hat{b}\hat{a}} & Q_{\hat{b}\hat{b}} \end{array}\right)\;$$
2. Mapping the real-valued ambiguity to integer with the integer estimator. The integer estimation procedure can be described as$\overset{⌣}{a}=I(\hat{a})$, with $I:{\mathbb{R}}^{n}\to {\mathbb{Z}}^{n}$.
3. Performing ambiguity acceptance test. The fixed integer $\overset{⌣}{a}$ is validated with ambiguity acceptance tests. If $\overset{⌣}{a}$ is rejected by the test, the procedure is finished and the float solution will be used as the final solution
4. Updating real-valued parameters by $\overset{⌣}{b}=\hat{b}-Q_{\hat{b}\hat{a}}Q_{\hat{a}\hat{a}}^{-1}(\hat{a}-\overset{⌣}{a})$ if $\overset{⌣}{a}$ is accepted by the ambiguity acceptance test.

The ambiguity acceptance test problem can be solved by either an integer aperture estimator or a hypothesis test. A typical hypothesis test problem involves three aspects: probability basis, a threshold determination approach and a test statistic. The integer aperture estimation for ambiguity acceptance test is based on the ambiguity residual distribution, which considers the stochastic property of the fixed integer candidate, thus provides a sound probability basis. The ambiguity residual is defined as [3,4]:

(3) $$\overset{⌣}{\varepsilon }=\hat{a}-\overset{⌣}{a},\overset{⌣}{a}\in {\mathbb{Z}}^{n},\hat{a}\in {\mathbb{R}}^{n}\;$$

The probability density function (PDF) of the ambiguity residual $\overset{⌣}{\varepsilon }$ is defined as [4]:

(4) $$f_{\overset{⌣}{\varepsilon }}(x)=\sum_{z\in {\mathbb{Z}}^{n}}f_{\hat{a}}(x+z)s_{0}(x),s_{0}(x)=\{\begin{array}{ll} 1 & \mathrm{if}\;x\in S_{0}\\ 0 & \mathrm{otherwise}\; \end{array}\;$$

where $S_{z}$ is the pull-in region of integer estimation centered at integer vector z; $f_{\hat{a}}(x)$ is the PDF of float ambiguity $\hat{a}$, which follows the multivariate normal distribution. $f_{\hat{a}}(x)$ can be calculated with:

(5) $$f_{\hat{a}}(x)=\frac{1}{\sqrt{\left|Q_{\hat{a}\hat{a}}\right|{\left(2\pi \right)}^{n}}}exp\{-\frac{1}{2}{\left\|x-a\right\|}_{Q_{\hat{a}\hat{a}}}^{2}\}\;$$

where |⋅| is the determinant of the matrix; exp{⋅} is the exponential operator. $\|\cdot {\|}_{Q_{\hat{a}\hat{a}}}^{2}$is the squared Mahalanobis distance operator [5,6], which can be calculated by ${\left(\cdot \right)}^{T}Q_{\hat{a}\hat{a}}^{-1}(\cdot )$.

Integer aperture (IA) estimation is a class of ambiguity estimator, which can map the real ambiguity into either integer or float solution. IA estimators allow for presence of gaps between the adjacent acceptance regions, so the integer estimation is only a special case of IA estimation [7,8]. According to the IA estimation theory, the probability of the ambiguity acceptance test outcomes can be calculated by [9]:

(6) $$\begin{array}{l} P_{s}=P(\bar{a}=a)=\int_{\Omega_{a}}f_{\hat{a}}(x)dx\\ P_{f}=P(\bar{a}\neq a)=\int_{\Omega \backslash \Omega_{a}}f_{\hat{a}}(x)dx=\sum_{z\neq a}\int_{\Omega_{z}}f_{\hat{a}}(x)dx=\int_{\Omega_{0}}\left(f_{\overset{⌣}{\varepsilon }}(x)-f_{\hat{a}}(x+a)\right)dx\\ P_{u}=P(\bar{a}=\hat{a})=1-P_{s}-P_{f} \end{array}$$

where $P_{s}$, $P_{f}$ and $P_{u}$ are the success rate, the failure rate and the undecided rate respectively. $\bar{a}$ is the output of an IA estimator. Comparing to the integer estimator, the IA estimator is a mixed estimator, since its outcomes can be either an integer vector or a real-valued vector. Ω and $\Omega_{a}$ are acceptance region space and a particular acceptance region centered at a. There are four types of threshold determination methods in ambiguity acceptance test: the empirical method, significance test, likelihood ratio approach and the fixed failure rate approach [10]. The empirical method can only provide a crude threshold but it is still the most popular way. The significance test approach is popular in early stages but it is not considered suitable for ambiguity acceptance test, since the ambiguity acceptance test cannot be treated as the standard hypothesis test problem. The likelihood ratio approach is a standard hypothesis test, but its performance in ambiguity acceptance test has not been systematically studied. The fixed failure rate (FF-) approach can control the probability of type II error, thus being considered promising but its computation burden hinders its application in real-time scenarios. In order to reduce the computation burden of the FF-approach, a number of methods have been proposed, such as the look-up table method [11,12] and the threshold function method [2,13,14]. 

The third aspect of the hypothesis test is about the test statistics of the ambiguity acceptance test, which has been the major focus of ambiguity acceptance test research. In early stages, many ambiguity acceptance tests have been derived from different perspective, such as the F-ratio test [15,16], the ratio test [17], the difference test [18], the projector test [19,20] and so forth. These test statistics are empirically efficient, although some of them are not theoretical rigorous [21]. Within the framework of the integer aperture estimation, the ellipsoidal integer aperture (EIA) [9], integer aperture bootstrapping (IAB) [22], integer aperture least-squares (IALS) [23], penalized integer aperture (PIA) [24] and optimal integer aperture (OIA) [25] were proposed. An extensive comparison between different integer aperture estimators has been made and the results indicate that the ratio test and the difference test are suboptimal estimator in terms of fixed failure rate [8,10]. A comparison among the OIA, the ratio test and the difference test indicates that the OIA is theoretically optimal, although it is not computationally efficient. Meanwhile, the ratio test and the difference test are efficient but suboptimal [26]. The difference test is a rough approximation of the OIA and can be generalized to bridge the gap between the difference test and the OIA. The generalized difference test employs information of multiple integer candidates, which makes the difference test more rigorous without significantly increasing the computation cost. 

In this study, we propose the generalized difference test (GDT), which improves the performance of the difference test by employing more than two integer candidates. The relationship between the difference test and the OIA and the GDT is also analyzed.

## 2. The Sub-Optimality of the Difference test

Currently, the most popular methods for the ambiguity acceptance tests include the ratio test and the difference test. Both employ two squared Mahalanobis distances for construction of test statistics with the difference in their test statistics formation. According to a previous comparison between the ratio test and the difference, the difference test is theoretically better than the ratio test [26,27], so we focus on the difference test in the remaining discussion. In this section, we compare the difference test and the OIA to reveal the sub-optimality of the difference test.

### 2.1. The Difference Test

The difference test is defined as [18]:

(7) $${\left\|\hat{a}-{\overset{⌣}{a}}_{2}\right\|}_{Q_{\hat{a}\hat{a}}}^{2}-{\left\|\hat{a}-\overset{⌣}{a}\right\|}_{Q_{\hat{a}\hat{a}}}^{2}\geq \mu_{D}\;$$

where $\overset{⌣}{a}$ and ${\overset{⌣}{a}}_{2}$ are known as the ’best integer candidate‘ and the ’second best integer candidate‘ respectively. The difference test uses the difference between two squared Mahalanobis distance as the test statistics. $\mu_{D}$ is the threshold for the difference test. In the integer least-squares estimation, the best integer candidate is defined as the integer vector has the shortest squared Mahalanobis distance to the float solution, which is expressed as:

(8) $$\overset{⌣}{a}=\;\arg\underset{z\in {\mathbb{Z}}^{n}}{\min}{\left\|\hat{a}-z\right\|}_{Q_{\hat{a}\hat{a}}}^{2},z\in {\mathbb{Z}}^{n}$$

Similarly, the second-best candidate has the second shortest squared Mahalanobis distance. The acceptance region of the difference test can be explicitly expressed and the difference test is a member of IA estimator, which is known as the difference test integer aperture (DTIA). Comparing to the OIA, DTIA only employs two integer vectors in the test statistics construction, while OIA involves infinite number of integer candidates.

The probability basis of the DTIA can be derived from the PDF of the float solution. It is noticed that the PDF of the multivariate normal distribution presents the exponential relationship with the squared Mahalanobis distance. Hence, the ratio of the two PDFs subject to the best and the second-best integers can be expressed as:

(9) $$\frac{f_{\hat{a}}(x+a-{\overset{⌣}{a}}_{2})}{f_{\hat{a}}(x+a-\overset{⌣}{a})}=\exp\left\{-\frac{1}{2}\left({\left\|x-{\overset{⌣}{a}}_{2}\right\|}_{Q}^{2}{}_{\hat{a}\hat{a}}-{\left\|x-\overset{⌣}{a}\right\|}_{Q}^{2}{}_{\hat{a}\hat{a}}\right)\right\}\;$$

where a is the true integer vector but it is unknown in reality. According to the definition of the IA estimator, the acceptance region of the IA estimators is ’z-translational invariant’, which means the shape of the acceptance region is independent from its center. The relationship expressed in Equation (9) also holds for any other integer vector offset. Applying the ’z-translational invariant‘ property again, we have:

(10) $$\frac{f_{\hat{a}}(x+a)}{f_{\hat{a}}(x-z_{2})}=\exp\left\{-\frac{1}{2}\left({\left\|x\right\|}_{Q}^{2}{}_{\hat{a}\hat{a}}-{\left\|x-z_{2}\right\|}_{Q}^{2}{}_{\hat{a}\hat{a}}\right)\right\}，z_{2}\neq 0\;$$

The equation shows that the difference test statistics lies in the ratio of the two PDFs; taken the log form of the equation, then we have:

(11) $${\left\|x-z_{2}\right\|}_{Q}^{2}{}_{\hat{a}\hat{a}}-{\left\|x\right\|}_{Q}^{2}{}_{\hat{a}\hat{a}}=2\log_{e}\left\{\frac{f_{\hat{a}}(x+a)}{f_{\hat{a}}(x-z_{2})}\right\}\;$$

The equation shows that the difference test is actually equivalent to the ratio of two PDFs.

Then substituting Equation (7) into Equation (9), we get another form of the difference test:

(12) $$\Omega_{\mathrm{DT}IA,0}=\{x\in S_{0}|\frac{f_{\hat{a}}(x-z_{2})}{f_{\hat{a}}(x+a)}\leq \exp\{-\frac{1}{2}\mu_{D}\}\}\;$$

The equation shows that the threshold of the test can be derived from $\mu_{D}$. 

Applying the ’z-translational invariant‘ property, the acceptance region of the difference test integer aperture (DTIA) estimator can also be expressed as:

(13) $$\Omega_{\mathrm{DT}IA,0}=\{x\in S_{0}|\frac{f_{\hat{a}}(x+a)}{f_{\hat{a}}(x-z_{2})}\geq \sqrt{e^{\mu_{D}}}\}\;$$

### 2.2. The Optimal Integer Aperture Estimation 

The optimal integer aperture (OIA) estimator is derived from a constrained optimization problem. It is optimal in sense of the maximum success rate with given failure rate constraint. In the ambiguity acceptance test problem, the success rate and the failure rate are both positively correlated to the acceptance region size. For a particular IA estimator, more optimistic threshold always means higher success rate and higher failure rate at the same time, In other word, it is impossible to pursue the highest success rate without increasing its failure risk. As a trade-off, one can strive for the highest success rate with certain failure rate constraint. The objective function of the constrained optimization problem in ambiguity acceptance test can be expressed as [25]:

(14) $$\max_{\Omega_{0}\subset S_{0}}P_{s}\;\mathrm{subject}\;\mathrm{to}:\;P_{f}\leq c\;$$

where $P_{s}$, $P_{f}$ are the success rate and failure rate of the IA estimator, which can be calculated with Equation (6); c is the failure rate tolerance.

The Neyman-Pearson lemma gives a solution to this constrained maximization problem [28]. The Neyman-Pearson lemma states that the constrained maximization problem

(15) $$\max_{\Omega \subset {\mathbb{R}}^{n}}\int_{\Omega }f(x)dx\;\mathrm{subject}\;\mathrm{to}:\;\int_{\Omega \subset {\mathbb{R}}^{n}}g(x)\mathrm{dx}=c\;$$

can be solved by:

(16) $$\Omega =\{x\in {\mathbb{R}}^{n}|f(x)\geq \lambda g(x)\},\lambda \in \mathbb{R}\;$$

where f(x) and g(x) are integrable functions over ${\mathbb{R}}^{n}$; λ is an unknown scalar to be determined. It is obvious that the parameter λ is connected to the constraint parameter c, although their relationship may not be explicit.

The problem solved by the Neyman-Pearson lemma is defined on ${\mathbb{R}}^{n}$ space, which cannot be directly applied to the ambiguity acceptance test problem. Then, the lemma was extended to the ’z-translational invariant‘ case and the extended Neyman-Pearson lemma can be used to solve the ambiguity acceptance test problem. The extended Neyman-Person lemma is given as [8,25]:

(17) $$\Omega =\{x\in {\mathbb{R}}^{n}|\sum_{z\in {\mathbb{Z}}^{n}}f(x+z)\geq \lambda \sum_{z\in {\mathbb{Z}}^{n}}g(x+z),\lambda \in \mathbb{R}\}\;$$

More specifically, the two integrable functions f(x) and g(x) in the ambiguity acceptance test problem can be expressed as:

(18) $$\Omega_{OIA,0}=\{x\in S_{0}|\sum_{z\in {\mathbb{Z}}^{n}}f_{\hat{a}}(x+z)\geq \lambda (\sum_{z\in {\mathbb{Z}}^{n}}f_{\hat{a}}(x+z)-f_{\hat{a}}(x+a)),z\in {\mathbb{Z}}^{n}\}\;$$

where $S_{0}$ is the pull-in region of integer estimator centered at 0. The equation shows that the OIA also can be written into the ratio of two PDFs form.

Considering the definition of the PDF of the ambiguity residuals, Equation (18) can be rewritten as:

(19) $$\Omega_{OIA,0}=\{x\in S_{0}|\frac{f_{\hat{a}}(x+a)}{f_{\overset{⌣}{\varepsilon }}(x)}\geq \mu_{O},\mu_{O}=\frac{\lambda -1}{\lambda }\}\;$$

where $\mu_{O}$ is the threshold of the OIA. Similar to the parameter λ, it also has implicit relationship with the failure rate tolerance c. The equation can also be equivalently expressed as:

(20) $$\Omega_{OIA,0}=\{x\in S_{0}|\frac{f_{\hat{a}}(x+a)}{\sum_{z\in {\mathbb{Z}}^{n}}f_{\hat{a}}(x+z)}\geq \mu_{O},\mu_{O}=\frac{\lambda -1}{\lambda }\}\;$$

There are two challenges in OIA implementation: (1) determining the threshold $\mu_{O}$ according to the failure rate tolerance, and (2) calculating the test statistics involving infinite number of PDF terms.

The first challenge comes from the implicit relationship between the threshold $\mu_{O}$ according to the failure rate tolerance. The implicit relationship can be numerically determined with the Monte-Carlo method and then the threshold can be determined by a root-finding algorithm. The detailed algorithm can be found in the studies [10,29]. The Monte-Carlo procedure is computation extensive, while it can be mitigated with the look-up table or threshold function method.

The second challenge is solved by an approximation method. As the float solution follows multivariate normal distribution, corresponding squared Mahalanobis distance follows $\chi^{2}(n)$ distribution. Since $\int_{0}^{\infty }\|x{\|}_{Q_{\hat{a}\hat{a}}}^{2}dx=1$, it is possible to find a finite radius R fulfilling the following requirement:

(21) $$\int_{0}^{R}\|x{\|}_{Q_{\hat{a}\hat{a}}}^{2}dx=1-\varepsilon \;$$

where ε is the probability tolerance controlling the implementation error. Smaller ε means the implementation is closer to the theory and consequently heavier computation burden. Then, the integers within the radius R are considered as the valid integer set and used to compute the $f_{\overset{⌣}{\varepsilon }}(x)$ [4,30,31]. The integer set can be defined as:

(22) $$\|z-\hat{a}{\|}_{Q_{\hat{a}\hat{a}}}^{2}\leq R,\forall z\in {\mathbb{Z}}^{n}\;$$

**Figure 1:**
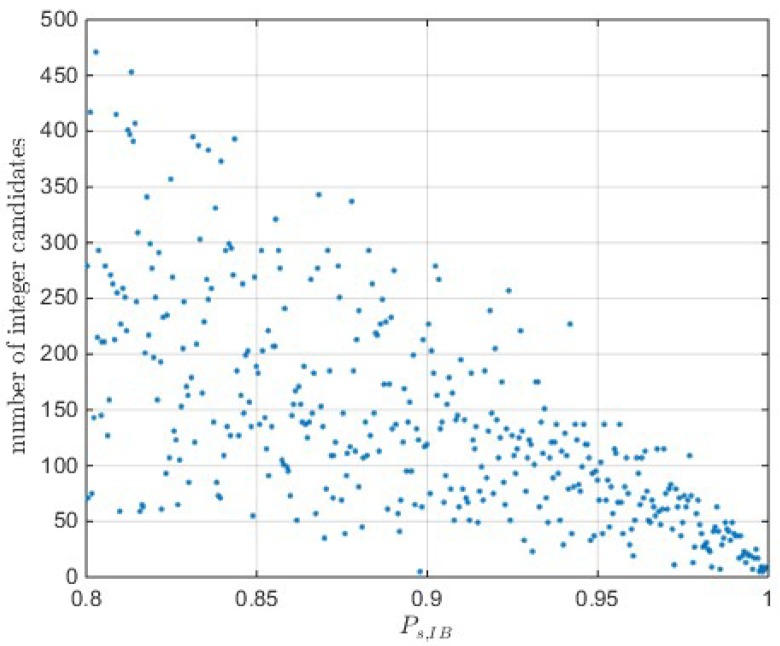
Number of integer candidates in the trust region with significance level.

The radius parameter R can be determined according to the cumulated distribution function (CDF) of the $\chi^{2}(n)$ distribution. An example of integer number in OIA implementation with the probability tolerance $\varepsilon =10^{-12}$ is presented in Figure 1. The figure shows that the integer number and its variability are dramatically decreases as the integer bootstrapping (IB) success rate increases. IB success rate serves as a tight lower bound of the integer least-squares (ILS) success rate and can present the underlying model strength. The weaker underlying model means the lower IB success rate. Therefore, the figure shows that the number of integer candidates is correlated to the underlying model strength. For most cases, the computation of OIA involves hundreds of integer candidates with the given significance level. A larger integer candidate set means more extensive computational burden. Therefore, computation complexity is the one of the major challenge of OIA in practice. 

### 2.3. The Discrepancy between the DTIA and the OIA

**Figure 2:**
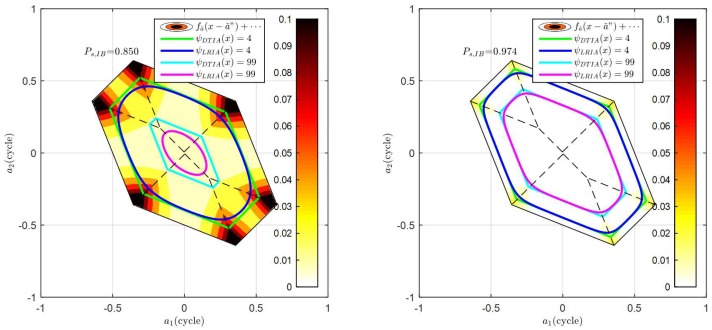
Demonstration of the acceptance region difference between the OIA and the DTIA with weak model (**left**) and strong model (**right**).

It is noticed that both the DTIA and the OIA can be expressed as the ratio of PDFs, while the difference is the denominator. Comparing Equation (13) and (20), it is realized the sub-optimality of the difference test is actually the result of only taking the PDF contribution of $z_{2}$ while OIA takes all $z\in {\mathbb{Z}}^{n}$. It can be anticipated that that the performance of the DTIA would be closer to OIA when the contribution of $z_{3},z_{4},\cdots$ are negligible. If the PDF contribution of the remaining integers, such as $z_{3},z_{4},\cdots$ in $f_{\overset{⌣}{\varepsilon }}(x)$ is not negligible, the performance of the difference test would be more distant from OIA. A two-dimensional demonstration of the acceptance region difference between the OIA and the DTIA is illustrated in Figure 2. The figure compares two scenarios with stronger underlying model and weaker underlying model, with their integer bootstrapping (IB) success rate 0.850 and 0.974 respectively. For each model, different thresholds are compared. The acceptance region of the OIA and the DTIA are given with the same λ. The figure also presents the PDF contribution of $z_{3},z_{4},\cdots$ in the ambiguity residual PDF in different colors. Dark color means more significant PDF contribution to $f_{\overset{⌣}{\varepsilon }}(x)$. For the two-dimensional case, the acceptance region of integer least-squares estimator (ILS) is typically a hexagon, which has 6 adjacent pull-in regions. The hexagon can be further divided into 6 parts, which corresponds to 6 different ’the second-best integer candidates’. The boundaries of these 6 parts are highlighted with the dash line. It is noticed that $\hat{a}$ falling in the region close to the dash line has similar Mahalanobis distance to the ‘second best integer candidate’ and the ‘third best integer candidate.’ Therefore, the value of $f_{\hat{a}}(x+z_{2})$ and $f_{\hat{a}}(x+z_{3})$ should be the similar, especially for the corners of the hexagon. However, DTIA only considers $f_{\hat{a}}(x)$ and $f_{\hat{a}}(x+z_{2})$, while $f_{\hat{a}}(x+z_{3})$ is not taken into consideration. Therefore, the most significant discrepancy between the acceptance region of DTIA and the OIA are the corner regions. The figure indicates that the PDF contribution of $z_{3},z_{4},\cdots$ in weaker model is more significant than the stronger model. Correspondingly, the acceptance region discrepancy between DTIA and OIA is more obvious in the weak model than the strong model. Moreover, the discrepancy also depends on the selection of λ. Stricter thresholds cause more significant discrepancies between DTIA and OIA. Generally, the DTIA is always over-optimistic than the OIA since the potential failure risk from $z_{3},z_{4},\cdots$ is not considered. The risk is negligible in the strong model, while it becomes more obvious as the underlying model becomes weaker. It concludes that only considering two integer candidates is not enough for a weak underlying model.

## 3. Generalized Difference Test

Above analysis indicates that the discrepancy between the DTIA and the OIA is caused by neglecting the probability contribution of $z_{3},z_{4},\cdots$. For the weak underlying model, the DTIA is not strict enough since it is over-optimistic to the failure risk. In this section, we proposed a generalized difference test approach, which can improve the performance of DTIA in case of weak underlying models.

### 3.1. Definition of the Generalized Difference test 

Current discrimination tests, such as the ratio test, the difference test, the projector test and a few other ambiguity acceptance tests, such as the model separability based ambiguity acceptance test approach [32] all employs the ’best integer candidate‘ and the ’second best integer candidate’. However, above analysis indicates that employing two integer candidates may not be enough for a weak model scenario. Therefore, we attempt to construct a new test statistic with more than two integer candidates.

The PDF of the ambiguity residuals can be expanded as:

(23) $$f_{\overset{⌣}{\varepsilon }}(x)=\sum_{z\in {\mathbb{Z}}^{n}}f_{\hat{a}}(x+z)=f_{\hat{a}}(x+a-z_{1})+f_{\hat{a}}(x+a-z_{2})+\cdots ,\forall x\in S_{0}\;$$

where $\left[z_{1},z_{2},\cdots ,z_{n}\right]$ are the best integer candidate, the second-best integer candidate to the nth best integer candidates, which are in decreasing ordered by their contribution to $f_{\overset{⌣}{\varepsilon }}(x)$. Then we have

(24) $$\frac{f_{\overset{⌣}{\varepsilon }}(x)}{f_{\hat{a}}(x+a)}=1+\frac{f_{\hat{a}}(x+a-z_{2})}{f_{\hat{a}}(x+a)}+\frac{f_{\hat{a}}(x+a-z_{3})}{f_{\hat{a}}(x+a)}+\cdots \;$$

Substituting Equations (10) into Equation (24), then the PDF ratio can be expressed by the difference test statistics, which is given as:

(25) $$\frac{f_{\overset{⌣}{\varepsilon }}(x)}{f_{\hat{a}}(x+a)}=1+\exp\left\{\frac{1}{2}\left({\left\|x\right\|}_{Q}^{2}{}_{{}_{\hat{a}\hat{a}}}-{\left\|x-z_{2}\right\|}_{Q}^{2}{}_{{}_{\hat{a}\hat{a}}}\right)\right\}+\exp\left\{\frac{1}{2}\left({\left\|x\right\|}_{Q}^{2}{}_{{}_{\hat{a}\hat{a}}}-{\left\|x-z_{3}\right\|}_{Q}^{2}{}_{{}_{\hat{a}\hat{a}}}\right)\right\}+\cdots \;$$

The equation allows for incorporating more than two integer candidates into the test statistics construction and each term has the difference test statistics form, which is denoted as the generalized difference test (GDT). The number of integer candidates depends on the underlying model strength. In order to discriminate the test statistics term number, the GDT with *m* integer candidates is denoted as GDT*m*. For example, the generalized difference test with three integer candidate case is defined as:

(26) $$\Omega_{GDT3,0}=\{x\in S_{0}|\exp\left\{\frac{1}{2}\left({\left\|x\right\|}_{Q}^{2}{}_{{}_{\hat{a}\hat{a}}}-{\left\|x-z_{2}\right\|}_{Q}^{2}{}_{{}_{\hat{a}\hat{a}}}\right)\right\}+\exp\left\{\frac{1}{2}\left({\left\|x\right\|}_{Q}^{2}{}_{{}_{\hat{a}\hat{a}}}-{\left\|x-z_{3}\right\|}_{Q}^{2}{}_{{}_{\hat{a}\hat{a}}}\right)\right\}\leq \frac{1}{\mu_{G3}}\}\;$$

In the equation, the second-best integer and the third best integer depends on the value of x.

The equation shows that the difference test is only a special case of the generalized difference test, which can be denoted as GDT2, the threshold of $\mu_{D}$ and $\mu_{G2}$ have following relationship:

(27) $$\mu_{D}=2log_{e}\mu_{G2}\;$$

However, this relationship only holds for GDT2. Another extreme case of the generalized difference test is the OIA, which can be denoted as GDT∞ since the OIA considers all integer candidates in the integer space ${\mathbb{Z}}^{n}$. 

### 3.2. The Acceptance Region of the GDT

**Figure 3:**
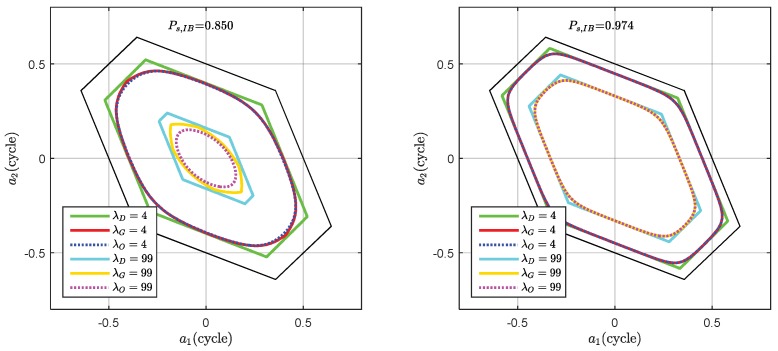
Comparison of the GDT3 acceptance region with the difference test and OIA acceptance region.

The generalized form of the difference test is also an integer aperture estimator. Its acceptance region is defined as:

(28) $$\Omega_{GDTm,0}=\{x\in S_{0}|\sum_{i=2}^{m}\exp\left\{\frac{1}{2}\left({\left\|x\right\|}_{Q}^{2}{}_{{}_{\hat{a}\hat{a}}}-{\left\|x-z_{i}\right\|}_{Q}^{2}{}_{{}_{\hat{a}\hat{a}}}\right)\right\}\leq \frac{1}{\mu_{Gm}}\}\;$$

where m is the term number of the GDT, which defines the number of term in the GDT test statistic. The GDT defines a class of scalable test statistics. Different test statistics correspond to different acceptance regions, which are bounded by the difference test and the OIA acceptance region. It allows the users to choose the optimal term number of GDT to balance between performance and computation burden. Generally, the GDT with more terms means heavier computation burden and closer to the OIA. The impact of incorporating both $z_{2}$ and $z_{3}$ in test statistics construction is demonstrated in Figure 3 . The figure shows the acceptance region of DTIA, GDT3 and the OIA with the weak model and strong model respectively. It is observed that the acceptance region of GDT3 is closer to the OIA than the difference test. The discrepancy between GDT3 and OIA is larger under weaker underlying model. For the same underlying model, the discrepancy also depends on the threshold settings. The discrepancy becomes more significant for a stricter threshold. For $\lambda_{O}=99$ case in the first model, the discrepancy between GDT3 and the OIA is significant, while the discrepancy for $\lambda_{O}=4$ case is less pronounced. For larger discrepancy cases, more integer candidates should be involved.

### 3.3. The Optimal Term Number of GDT

**Figure 4:**
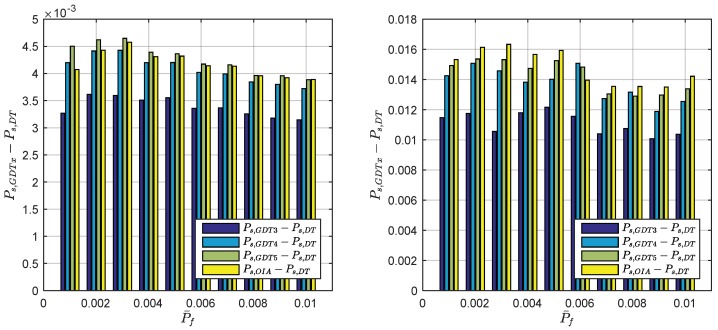
The impact of GDT term number on success rate subject to different failure rate tolerance: Mean value (**left**) and maximum value (**right**).

According to the previous analysis, optimal determination of the term number of GDT is meaningful to achieve good balance between the performance and the computation burden. Considering the computation burden, it makes good sense to select fewer integer sets that play the most important role in $f_{\overset{⌣}{\varepsilon }}(x)$ computation. The contribution of PDFs depends on the Mahalanobis distance, so we need search the best *m* sets of integer candidates first. In order to optimally determine the practical GDT term number, the impact of the integer candidate number on the success rate with fixed failure rate is examined by comparing the success rates between the OIA and the difference test, GDT3, GDT4 and GDT5. The simulation results are presented in Figure 4. The simulation employs about 400 examples with their IB success rate varying between 99.95% and 80%. The underlying model with IB success rate lower than 80% is considered too weak for ambiguity resolution. 100,000 samples are used in the Monte Carlo simulation to numerically determine the threshold, the success rate and the failure rate for each example. The left panel presents the average success rate difference between GDT and OIA, while the right panel shows the maximum success rate difference. The average success rate difference between the difference test and the OIA is about 0.3~0.5% and the maximum value reaches 1.6%. GDT3, GDT4 and GDT5 achieves higher success rate than the difference test but their improvements are not the same. The third best integer candidate $z_{3}$ contributes more than 74% out of all success rate improved by incorporating all integers. $z_{4}$ contributes about 17.5% and $z_{5}$ contributes about 3.7% on average. All the remaining integers contribute to about 3.6% success rate improvement on average. Overall, GDT3 yields the maximum profit and is recommended for the IB success rate higher than 80% case. For these users who do not care about computation resources, employing GDT4 or even GDT5 still further improves the success rate. 

### 3.4. Rapid Determination of the Threshold of the GDT

The fixed failure rate (FF-) approach is theoretically rigorous and applicable to all IA estimators but it is computation exhaustive. Due to the extensive computation in the Monte-Carlo procedure, the FF-approach is difficult to be applied in real-time application. In order to improve the computation efficiency, the lookup table method [11,12] and the threshold function method [2,13] have been proposed. The look-up table method is applicable to all IA estimators but it is model-dependent. Users are encouraged to create their own look up table according to the specific underlying model. The threshold function method is independent from ambiguity dimension and underlying function model and it was proposed for the difference test. Although this method is also attempted in the ratio test but it becomes more complex than the difference test case [33]. The method to mitigate computation burden for OIA has not been systematically studied.

According to the definition, the GDT is an extended form of the difference test, so we attempt to use the similar threshold determination method to improve its computation efficiency. The threshold function method has shown to work well for the difference test, so it is also worth to try in the GDT threshold determination. In order to applying a similar rational function as the difference test, the logarithm form of the GDT threshold is used. The relationship between fixed failure rate threshold of GDT3 and IB success rate can be given as:

(29) $$\log\mu_{G3}(x)=\frac{e_{1}+e_{2}x}{1+e_{3}x+e_{4}x^{2}}\;$$

where $e_{1},e_{2},e_{3},e_{4}$ are the model coefficients; x is the IB success rate, which can be calculated with $Q_{\hat{a}\hat{a}}$ [34,35]:

(30) $$P_{s,IB}=\prod_{i=1}^{n}(2\Phi (\frac{1}{2\sigma_{{\hat{a}}_{i|I}}})-1)\;$$

where Φ(⋅) is the cumulative distribution function (CDF) of the normal distribution. $\sigma_{{\hat{a}}_{i|I}}$ is thei th conditional variance subject to {1,⋯,i−1}. The conditional variance $\sigma_{{\hat{a}}_{i|I}}$ can be obtained by the LDL decomposition. The decorrelation procedure has to be applied since only decorrelated IB success rate is a tight lower bound of integer least-squares success rate [36]. The decorrelation method and the LDL decomposition method are described in the work [37].

**Figure 5:**
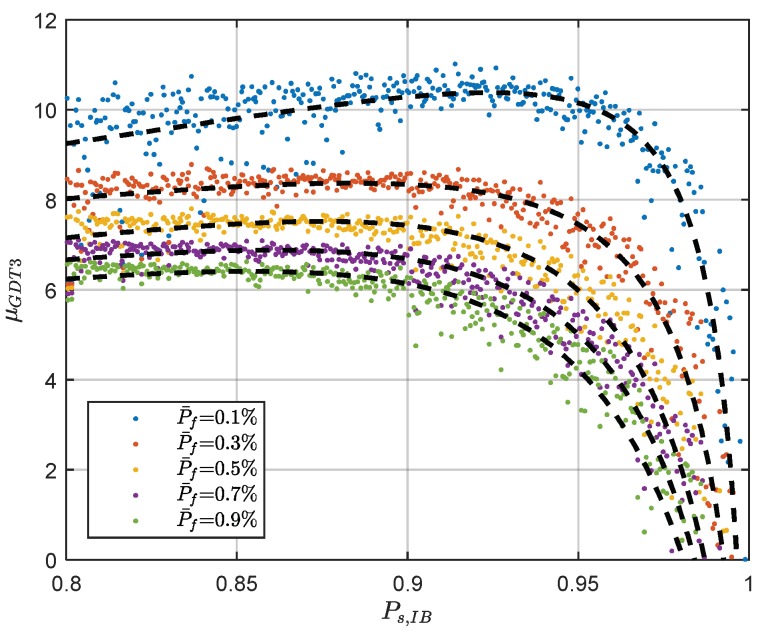
The threshold function of the GDT3.

The relationship between the logarithm GDT3 threshold and the IB success rate is illustrated in Figure 5. The figure indicates that the relationship between the logarithm of GDT3 threshold and the IB success rate is quite similar as the difference test threshold function. The Levenberg-Marquardt curve fitting algorithm is used to find the functional relationship [38,39] and the fitted curve is marked as the black dash line in the figure. The color dots are the threshold of GDT3 determined with the Monte-Carlo simulation method. The figure shows that the fitted curves agree with the observed threshold well. The impact of curve fitting on the success rate, failure rate and threshold for the difference test has been carefully examined in Reference [2] and GDT has quite similar performance, so it is not discussed in details. The coefficients of the threshold function for different failure rate tolerance is listed in Table 1. It is noticed the fitted curve for GDT is the logarithm form of the threshold, rather than the threshold. 

### 3.5. The Procedure of Applying GDT

After the term number and the threshold function are determined, the procedure for how to apply GDT in practice may be outlined. Recalling the four-step procedure to solve the mixed integer linear problem, the second and the third step should be redefined. The procedure of applying GDT can be summarized as follows:

1. Finding the best *m* integer candidates. The optimal integer estimator, the integer least-squares, can intermediately give an arbitrary number of best integer candidate sets. Hence, getting *m* best sets of integer candidates is just a sorting procedure.
2. Constructing the test statistics of GDT. For example, the test statistics of GDT3 can be computed as:
  (31) $${\hat{\mu }}_{GDT3}=\exp\left\{\frac{1}{2}\left({\left\|\hat{a}-\overset{⌣}{a}\right\|}_{Q}^{2}{}_{{}_{\hat{a}\hat{a}}}-{\left\|\hat{a}-{\overset{⌣}{a}}_{2}\right\|}_{Q}^{2}{}_{{}_{\hat{a}\hat{a}}}\right)\right\}+\exp\left\{\frac{1}{2}\left({\left\|\hat{a}-\overset{⌣}{a}\right\|}_{Q}^{2}{}_{{}_{\hat{a}\hat{a}}}-{\left\|\hat{a}-{\overset{⌣}{a}}_{3}\right\|}_{Q}^{2}{}_{{}_{\hat{a}\hat{a}}}\right)\right\}\;$$
  where ${\hat{\mu }}_{GDT3}$ denotes for the computed test statistics of GDT3. $\overset{⌣}{a}$, ${\overset{⌣}{a}}_{2}$, ${\overset{⌣}{a}}_{3}$ are the best, second best, third best sets of integer candidates. The squared Mahalanobis distances can also be obtained with the integer least-squares searching procedure.
3. Computing IB success rate with Equation (26), then determining the threshold $log\mu_{GDT3}$ of GDT using the threshold function with specified IB success rate and failure rate tolerance. The threshold is a non-negative value, so the threshold is set to be zero if the computed threshold $log\mu_{GDT3}$ is negative.
4. Performing ambiguity acceptance test. If ${\hat{\mu }}_{GDT3}\leq \frac{1}{e^{\mu_{GDT3}}}$, or equivalently $\log(1/{\hat{\mu }}_{GDT3})\geq \mu_{GDT3}$, then the best integer candidate $\overset{⌣}{a}$ can be accepted by the ambiguity acceptance test, otherwise, reject it. Since the threshold function gives $log\mu_{GDT3}$, it is necessary to recover the $\mu_{GDT3}$ with an exponential function.

## 4. Performance Evaluation of the GDT

The performance of GDT can be evaluated in terms of success rate and percentage identical to OIA. The success rate acts as an important performance indicator for the IA estimation performance, the IA estimator achieves higher success rate subject to the given failure rate tolerance is preferred. The percentage identical to OIA is used to assess the optimality of the IA estimator.

Since the success rate of the GDT is difficult to compute analytically, the Monte Carlo approach is adopted. The description of success rate computation method using Monte Carlo method can be found in Reference [34]. About 400 samples with their IB success rates vary from 0.8 to 0.995 are selected to address the relationship between GDT performance and the underlying model. Since the discrepancy between DTIA and GDT is IB success rate sensitive, the comparison is made on group basis. These samples are divided into four groups with their IB success rate within [0.96,0.999], [0.92,0.959], [0.88,0.919], [0.84,0.879] and [0.80,0.849]. 

**Figure 6:**
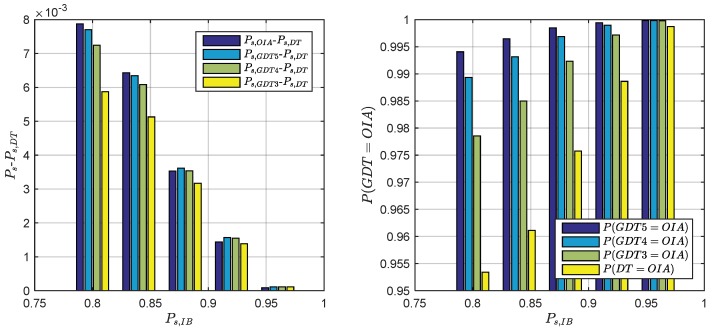
Performance comparison of GDT in terms of success rate gain against DT (**left**) and percentage identical to OIA (**right**).

The comparison results are presented in Figure 6. The left panel shows the average success gain of OIA and GDT with respect to DTIA. The results show that the success rate gain with respect to the difference test vanishes as the IB success rate increases. For $P_{s,IB}\geq$ 0.96 case, all tests achieve almost the same success rate as the OIA. In case of $P_{s,IB}\approx 0.8$ case, the average success rate between the difference test and the OIA increases 0.8%. The discrepancy between the GDT3 and difference tests also increases to about 0.6%. GDT3 accounts for more than 70% of the total possible success rate improvement, which is consistency to the previous analysis. Considering more integers also contribute to the success rate improvement, however, less significant. The figure also indicates that the discrepancy between GDT3 and GDT4 also increased as the IB success rate decreases, which means GDT3 may not be enough for even weaker underlying model. The success rate discrepancy between GDT3 and GDT4 achieves 0.13% for $P_{s,IB}\approx 0.8$ case. For these success rata critical applications, the impact of the fourth best integer candidate also should be considered. For the model with IB success rate lower than 0.8, maybe GDT4 or even GDT5 should be considered. 

The percentage identical to OIA is used to evaluate the optimality of the GDT estimators. The simulation results are presented in the right panel of Figure 6. The results show that the optimality of GDT increases as the IB success rate increases. For $P_{s,IB}>95\%$ case, the difference test achieves 99.9% identical to the OIA. However, the difference test becomes less and less optimal as the $P_{s,IB}$ decreases. For $P_{s,IB}=80\%$ case, the difference test only has 95.3% chance to make the same decision as the OIA but this percentage is improved to 97.8% by GDT3 and 99.4% by GDT5 respectively. For the case of IB success rates higher than 0.9, GDT3 achieves the percentage of higher than 99% identical to OIA. 

Comparing the two panels, it is noticed that the percentage identical to OIA improvement is more significant than the success rate improvement. This is because the percentage identical to OIA is used to measure the similarity of the GDT acceptance region to OIA acceptance region, rather than the success rate, while the acceptance region of OIA gives a good separation on failure probability and the success probability. 

## 5. Numerical Results from GNSS Baseline Data

**Figure 7:**
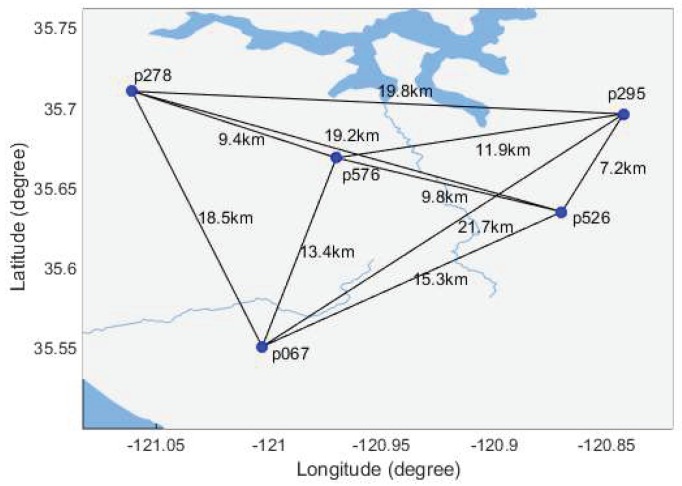
The distribution of the CORS stations.

The performance of the GDT is validated by epoch-by-epoch RTK processing of GNSSdata from the NGS CORS network, which is publicly accessible by the FTP server (ftp://geodesy.noaa.gov). As shown in Figure 7, the inter-station distances are all within 20 km. The observations collected on 1–7 June 2017 (DOY 152–158) are used in data analysis. Ten baselines for formed and processed epoch by epoch with the carrier phase ambiguity resolved by the LAMBDA approach [1]. The ambiguity resolved with the coordinate constrained model is used as the true integer ambiguity. The data analyzed with various GDT schemes are processed and the estimated integer ambiguities are compared with the true ambiguity to obtain the empirical success rate. The threshold of the difference test and the GDT3 are determined with the threshold function method.

Since the difference test and GDT are all PDF based test statistics, it is critical to establishing a realistic stochastic model for the observations. The stochastic modeling in the data processing can be described as follows:

1. The ionosphere weighted model is used to capture the ionosphere biases. The elevation dependent and baseline dependent stochastic model is used for the priori ionospheric noise. With strong priori ionosphere constraint, the short-baseline is equivalent to the ionosphere-fixed model.
2. The elevation dependent weighting model is used to reflect the observation noise.
3. The posterior variance factor is estimated on epoch basis to adapt the temporal variation of observation noise. Since the difference test and GDT are sensitive to the variance factor, so capture the temporal variation of variance factor is important. A more detailed stochastic modeling strategies can be found in the work [14].

After the integer ambiguities are estimated with the ILS estimator, the test statistics of the difference test and the GDT3 are computed and their thresholds are computed according to the IB success rate and threshold function. The obtained integer solutions are compared with the true ambiguity integers to calculate the actual success rate and failure rate, which are referred as the empirical success rate and empirical failure rate respectively.

**Figure 8:**
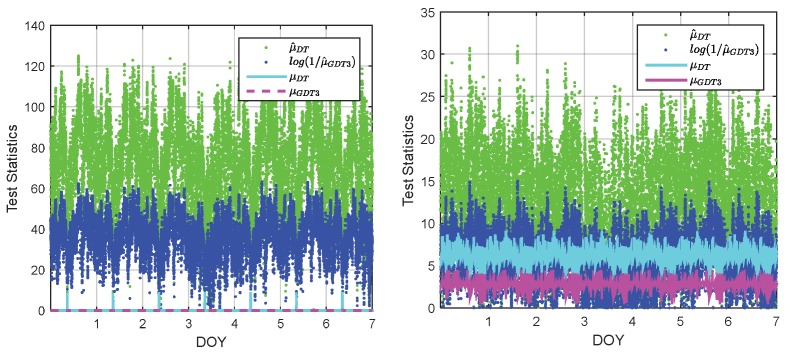
Illustration of DTIA and GDT3 test statistics and FF-threshold for baseline P256-P259 (**left**) and P067-P259 (**right**).

In order to give a more intuitive comparison between DTIA and GDT3, their test statistics and thresholds are compared. The two baselines, P526-P295 and P067-P295 are chosen since they are the shortest and the longest baseline in the network respectively. The test statistics and the FF-threshold calculated from the threshold function are shown in Figure 8. For the convenience, the modified form of GDT3 is used in the comparison. The figures indicate that the amplitude of the GDT3 test statistics is generally smaller than that of the DTIA time series, while the corresponding threshold is also lower than that of the DTIA threshold. The value of GDT3 test statistics is reduced as the baseline length increases, while the threshold increases as the baseline length increases and consequently, the long baseline case has lower fix rate for both DTIA and GDT3. According to the threshold function, a higher value in the threshold function generally means lower success rate.

**Figure 9:**
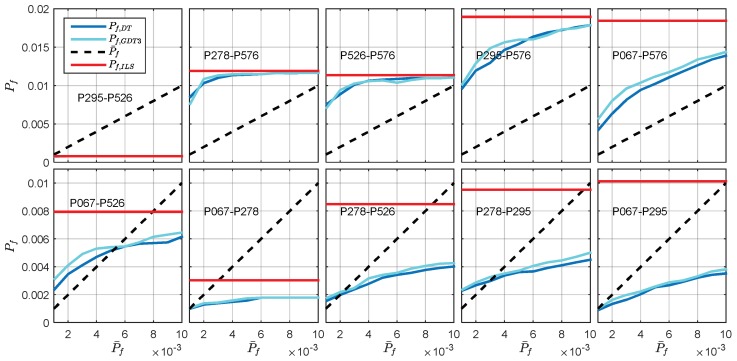
Comparison of the actual failure rate of DT and GDT3 against the failure rate tolerance.

DTIA and GDT are compared by means of the empirical success rate and empirical failure rate. The empirical failure rate of the difference test and GDT3 for all baselines is presented in Figure 9. The results are sorted in ascending order according to their baseline length. The ILS failure rate of the baselines and the failure rate tolerance ${\bar{P}}_{f}$ are also presented in the figure. The ILS failure rate results stay as the upper bound of the empirical failure rate since no quality control is applied in this case. The results demonstrate that both the difference test and the GDT3 can efficiently reduce the failure risk. For most baselines, the empirical failure rate of GDT3 is quite similar to the DTIA and some of them are slightly higher than the difference test, For the lower panels, both the difference test and GDT3 can meet the failure rate tolerance, while the empirical failure rate for baseline P278-P576, P526-P576, P295-P576 and P067-P576 do not fit with the threshold well. It is noticed that all these baselines are connected to the P576 station, so the unexpected large failure rate may be caused by unexpected biases or mismodeling of the station-specified observation noise. Generally, the failure rate achieved by the threshold function of GDT remains comparable with that of the difference test.

**Figure 10:**
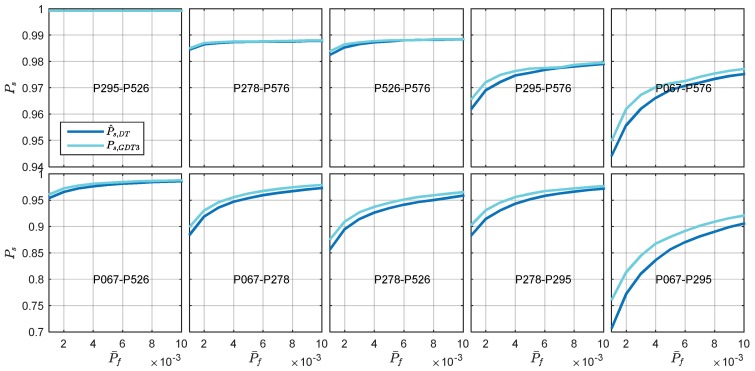
Illustration of the empirical success rates of DT and GDT3 test statistics.

The empirical success rates of the difference test and GDT3 are presented in Figure 10. It is observed that for a particular baseline, the success rate increases as the failure rate tolerance getting larger. The empirical success rate also decreases as the baseline length increases. Generally, the GDT3 achieves higher success rate than the difference test for all baselines. Since the baselines are sorted by their baseline length, the figures also indicate that the success rate of the baselines decreases as the baseline length increases, while the failure rate does not have similar results. The improvement achieved with GDT3 is more significant for the weaker underlying model. For example, the empirical success rate of GDT3 is about 7% higher than the difference test, although they have similar empirical failure rate. 

## 6. Concluding Remarks

A generalized difference test (GDT) approach for GNSS ambiguity acceptance test has been investigated in this study. Current discrimination tests, such as the ratio test, the difference test, only consider two integer candidates in the construction of test statistics. In this work, we examined the role of the third, fourth and fifth best integer candidates in ambiguity acceptance test and their contribution to the ambiguity residual PDF is analyzed. It concludes that considering two integer candidates may underestimate the failure risk, especially for a weak underlying model. In order to improve the performance of the difference test, a generalized difference test (GDT) employing an arbitrary number of integer candidates is proposed. The simulation results have indicated that considering more sets of integer candidates in test statistics construction can improve the success rate and the percentage identical to OIA. 

Comparing to other GDTs, GDT3 contributes to more than 70% of the success rate gain with only considering one extra integer candidates. Therefore, GDT3 is considered as a first choice to balance between the performance and computation complexity when the integer bootstrapping success rate higher than 80% cases. However, more sets of integer candidates may be involved for even weaker underlying models to achieve higher performance. Similar to the difference test the rational function is constructed to rapidly determine the fixed failure rate threshold for the GDT3, which helps circumvent the time-consuming Monte-Carlo procedure and significantly improves the computation efficiency of GDT3. The performance of GDT3 is validated with real GNSS datasets processed in single epoch RTK mode. The test results indicate that the GDT3 achieves higher empirical success rate while the empirical failure rate remains comparable as the difference test in all tested baselines. The improvement provided by the GDT3 is more significant for the longer baseline case. For the 20km baseline, the empirical success rate of GDT3 gains 7% if the difference test and the GDT3 failure rate remains comparable.

## Figures and Tables

**Table 1:** The coefficient of the threshold function for the GDT3 with different failure rate tolerance.

${\bar{P}}_{f}(\%)$	$e_{1}\;$	$e_{2}\;$	$e_{3}\;$	$e_{4}\;$
0.1	1.6691	−1.6660	−1.7798	0.7849
0.2	1.6957	−1.6990	−1.7434	0.7492
0.3	1.6277	−1.6333	−1.7419	0.7491
0.4	1.5834	−1.5899	−1.7383	0.7471
0.5	1.4711	−1.4775	−1.7617	0.7723
0.6	1.4260	−1.4314	−1.7653	0.7780
0.7	1.3723	−1.3796	−1.7740	0.7875
0.8	1.3850	−1.3959	−1.7589	0.7726
0.9	1.2930	−1.3041	−1.7851	0.8001
1.0	1.2639	−1.2771	−1.7881	0.8035
